# Household Survey Measurement of Newborn Postnatal Care: Coverage, Quality Gaps, and Internal Inconsistencies in Responses

**DOI:** 10.9745/GHSP-D-21-00209

**Published:** 2021-12-31

**Authors:** Kimberly Peven, Louise Tina Day, Debra Bick, Edward Purssell, Cath Taylor, Joseph Akuze, Lindsay Mallick

**Affiliations:** a Florence Nightingale Faculty of Nursing, Midwifery & Palliative Care, King's College London, London, United Kingdom.; b Maternal and Newborn Health Group, Department of Infectious Disease Epidemiology and Centre for Maternal, Adolescent, Reproductive, & Child Health (MARCH), London School of Hygiene & Tropical Medicine, London, United Kingdom.; c Warwick Clinical Trials Unit, University of Warwick, Coventry, United Kingdom.; d Little Havens Children's Hospice, Benfleet, United Kingdom.; e School of Health Sciences, University of Surrey, Guildford, United Kingdom.; f Department of Health Policy, Planning and Management and Centre of Excellence for Maternal, Newborn and Child Health, Makerere University School of Public Health, Kampala, Uganda.; g Department of Family Science, School of Public Health, University of Maryland, College Park, MD, USA.; h Avenir Health, Glastonbury, CT, USA.

## Abstract

**Background:**

Reliable measurement of newborn postnatal care is essential to understand gaps in coverage and quality and thereby improve outcomes. This study examined gaps in coverage and measurement of newborn postnatal care in the first 2 days of life.

**Methods:**

We analyzed Demographic and Health Survey data from 15 countries for 71,366 births to measure the gap between postnatal contact coverage and content coverage within 2 days of birth. Coverage was a contact with the health system in the first 2 days (postnatal check or newborn care intervention), and quality was defined as reported receipt of 5 health worker-provided interventions. We examined internal consistency between interrelated questions regarding examination of the umbilical cord.

**Results:**

Reported coverage of postnatal check ranged from 13% in Ethiopia to 78% in Senegal. Report of specific newborn care interventions varied widely by intervention within and between countries. Quality-coverage gaps were high, ranging from 26% in Malawi to 89% in Burundi. We found some internally inconsistent reporting of newborn care. The percentage of women who reported that a health care provider checked their newborn's umbilical cord but responded “no” to the postnatal check question was as high as 16% in Malawi.

**Conclusion:**

Reliable measurement of coverage and content of early postnatal newborn care is essential to track progress in improving quality of care. Postnatal contact coverage is challenging to measure because it may be difficult for women to distinguish postnatal care from intrapartum care and it is a less recognizable concept than antenatal care. Co-coverage measures may provide a useful summary of contact and content, reflecting both coverage and an aspect of quality.

## Abstract (precis)

Reliable measurement of postnatal content of care is currently lacking despite the critical importance of care in this vulnerable period. We found that there is a large quality-coverage gap with missed opportunities for quality care as well as internal inconsistencies in responses to newborn questions.

## Teaser

**Key Findings:**

- Reports of receipt of a newborn postnatal care check do not necessarily reflect adequate provision of interventions resulting in large gaps between reported contact coverage and recommended content of newborn postnatal care (26%–89%) in 15 low- and middle-income countries.
- We found internal inconsistencies in survey responses regarding receipt of newborn postnatal care (“no” to whether anyone checked on the newborn's health, but “yes” to questions on specific newborn interventions), which were as high as 16% in Malawi.

**Key Implications:**

- Co-coverage measures may provide a useful summary of contact and content, reflecting coverage and an aspect of quality for tracking and monitoring progress towards global goals.
- Researchers can use this individual-level measure for equity analyses and to easily carry out multicountry studies or time-trend studies.
- Gaps in the provision of newborn care interventions indicate missed opportunities for delivering high-quality postnatal care. Facility managers and policy makers should identify bottlenecks or gaps in service provision that can be addressed to improve the quality of care.

## INTRODUCTION

Progress in reducing neonatal mortality has been slower than progress in reducing older child mortality^1^ despite the availability of evidence-based interventions that could reduce deaths.^2^ Most newborn deaths occur in the first 2 days of life, so universal coverage of high-quality postnatal care is critical.^3^^,^^4^ After a facility birth, current recommendations are for healthy mother/newborn dyads to be cared for in facilities for the first 24 hours. After homebirth, the first postnatal contact is recommended to occur as soon as possible within 24 hours of birth.^5^ Recommended interventions include physical assessment, counseling of the family on danger signs in the newborn, support for breastfeeding, cord care, delayed bathing, appropriate clothing, encouragement of communication and play with the newborn, and promotion of infant vaccination.^5^

Reliable measurement of postnatal content of care is currently lacking despite the critical importance of care in this vulnerable period. Currently, the global tracking indicator for newborn postnatal care focuses on postnatal contact (e.g., postnatal check)^6^^,^^7^ without tracking whether those checks included recommended interventions.^8^ However, there is increasing interest in tracking content and quality of care over care contacts.^9^

Newborn health coverage data are increasingly available as the focus on newborns in global accountability frameworks has increased,^10^ yet gaps in newborn quality of care data persist. To better understand the quality-coverage gap, recent efforts to develop and investigate measures of effective coverage are underway. However, calculation of this measure often depends on the availability of both population-based survey data linked to health facility data or other quality of care data.^11^^–^^13^ As health facility surveys with publicly available data are not widely collected, proxies from survey data are often used. Measurement of quality of care in population-based surveys has typically used proxies of contact coverage, timeliness, and skill level of the health care provider.^14^ However, it is well established that reported contact with the health system is not indicative of receipt of adequate quality of care. In antenatal care measurement, the gap between contact with the health system and delivery of a comprehensive set of recommended interventions for antenatal care has been described by Hodgins and D'Agostino^15^ as the “quality-coverage gap.” This gap has also been shown for maternal and newborn postnatal care where across 17 countries, 65% of women/newborns had a skilled attendant at birth but only 3% received a total of 7 specific postnatal care interventions and practices.^16^ Additional questions on provider-initiated interventions for newborns added to the Demographic and Health Survey Program (DHS) core questionnaire in 2015 provide an opportunity to further evaluate the quality-coverage gap for newborns.^17^

Newborn health coverage data are increasingly available owing to increased focus on newborns in global accountability frameworks, yet gaps in newborn quality of care data persist.

National and international tracking relies on contact coverage indicators to guide policy, assess success, and inform service redesign, despite little assessment of the reliability of standard measures.^18^ Qualitative research exploring women's understanding of survey questions about receipt of a postnatal check for their newborn found that many did not understand what was meant by a postnatal “health check.”^19^ Postnatal check is commonly used as a proxy for receipt of newborn care at the population level. Given these recently added questions asked about postnatal care interventions, we can examine a proxy for the quality-coverage gap using a household survey-based measure of co-coverage (an index summing the total number of interventions received by a newborn out of a specified set of interventions) for postnatal care and examine internal consistency in responses to newborn-related questions.

In this article, we examine the concordance between the global postnatal care indicator—reported receipt of a newborn postnatal check (A)—and reported receipt of specific newborn care interventions (B) using nationally representative DHS surveys in sub-Saharan Africa and South Asia (Box). Specific aims included:

1. Describe survey-reported coverage of newborn postnatal check (A) and specific newborn care interventions and gaps in quality (B)
2. Describe internal consistency in survey-reported postnatal checks (A) and specific newborn care interventions (B)

BOX **Definitions of Terms**

**Newborn postnatal check (contact coverage):** Coverage of a newborn postnatal health check in the first 2 days of life (“yes” response to the survey question defined in Table 1 row A).

**Specific newborn care intervention coverage (content coverage):** Coverage of specific newborn care interventions in the first 2 days of life (“yes” response to survey questions in Table 1 rows B1–B5: B1, umbilical cord check; B2, temperature measurement; B3, counseling on danger signs in the newborn; B4, breastfeeding counseling; and B5, observation of breastfeeding).

**Co-coverage:** An index of the number of specific newborn care interventions received (sum of “yes” responses to survey questions in Table 1 rows B1–B5: B1, umbilical cord check; B2, temperature measurement; B3, counseling on danger signs in the newborn; B4, breastfeeding counseling; and B5, observation of breastfeeding).

**Any contact with a health care provider:** Coverage of either a newborn postnatal check (A) AND/OR any of the specific newborn care interventions (B1–B5) in the first 2 days of life.

**Quality-coverage gaps:**

1. **Intervention-specific quality-coverage gap:** The difference between any contact with a health care provider and coverage of a specific newborn care intervention (100 minus the percentage with intervention coverage, among those with any contact with a health care provider).
2. **Full content quality-coverage gap:** Any contact with a health care provider but not complete content coverage of all specific newborn care interventions.

**Internal inconsistency:** Newborns with a reported umbilical cords check (B1) and no reported postnatal check (A) (Table 3).

## METHODS

We included data from recent DHS surveys (2015–2018) in low- and lower middle-income countries in sub-Saharan Africa and South Asia. Countries were included if the questionnaire wording for postnatal checks and newborn care interventions matched the DHS7 core questionnaire wording shown in Table 1. The countries, years of the survey, number of women interviewed, and number of births in the 2 years before the survey, are shown in Table 2. DHS surveys are nationally representative, cross-sectional surveys using a standard core questionnaire that is comparable across countries and over time. Surveys are conducted with women of reproductive age (15–49 years) and collect important health and demographic information, including women's detailed birth histories. The DHS Program introduced a general newborn postnatal check question to their standard questionnaire in the fifth phase of the project (2003–2008).^21^^,^^22^ In the seventh phase of the project, 5 further questions were added about specific health care provider-initiated interventions for newborns in the first 2 days of life.^17^

**TABLE 1.** Postnatal Care Intervention Survey Questions, Postnatal Care Interventions, and Question Wording From the DHS-7 Core Questionnaire^17^

	Intervention	Question
A	Postnatal check	For facility births, women are asked about a newborn postnatal check while they were still in the facility. Later, they are asked separately about a newborn postnatal check after they left the facility. A postnatal check is counted if they report a check in the facility or after.
		438. Now I would like to talk to you about checks on (NAME)'s health after delivery—for example, someone examining (NAME), checking the cord, or seeing if (NAME) is OK. Did anyone check on (NAME)'s health while you were still in the facility?
		445. I would like to talk to you about checks on (NAME)'s health after you left (FACILITY IN 430). Did any health care provider or a traditional birth attendant check on (NAME)'s health in the two months after you left (FACILITY IN 430)?
		For non-facility births, women are asked about a newborn postnatal check more generally.
		453. I would like to talk to you about checks on (NAME)'s health after delivery—for example, someone examining (NAME), checking the cord, or seeing if (NAME) is OK. In the two months after (NAME) was born, did any health care provider or a traditional birth attendant check on (NAME)'s health?
B1	Umbilical cord check	457 a) During the first two days after (NAME)'s birth, did any health care provider do the following: Examine the cord?
B2	Temperature measurement	457 b) During the first two days after (NAME)'s birth, did any health care provider do the following: Measure (NAME)'s temperature?
B3	Danger sign counseling	457 c) During the first two days after (NAME)'s birth, did any health care provider do the following: Counsel you on danger signs for newborns?
B4	Breastfeeding counseling	457 d) During the first two days after (NAME)'s birth, did any health care provider do the following: Counsel you on breastfeeding?
B5	Breastfeeding observation	457 e) During the first two days after (NAME)'s birth, did any health care provider do the following: Observe (NAME) breastfeeding?

Abbreviation: DHS, Demographic and Health Survey.

**TABLE 2.** Included Countries, Survey Year, and Sample From Demographic and Health Surveys on Postnatal Checks and Newborn Care Interventions

Country	Survey Year	Number of Women Interviewed^a,b^	Number of Last (Most Recent) Births in the 2 Years Before the Survey^a^
Benin	2017–2018	15,928	5,390
Burundi	2016–2017	17,269	5,358
Cameroon	2018	13,527	3,843
Ethiopia	2016	15,683	4,221
Guinea	2018	10,874	2,948
Malawi	2015–2016	24,562	6,567
Mali	2018	10,519	4,075
Nepal	2016	12,862	1,958
Nigeria	2018	41,821	12,616
Pakistan	2017–2018	12,264	3,855
Senegal	2017	16,787	4,401
Tanzania	2015–2016	13,266	4,091
Uganda	2016	18,506	5,781
Zambia	2018	13,683	3,845
Zimbabwe	2015	9,955	2,417
Total		247,506	71,366

a Weighted.

b From ICF International.^20^

We focused our analysis on the postnatal check (A) and 5 health care provider-initiated specific newborn care interventions included in the standard DHS questionnaire (B), namely, the following: B1, umbilical cord check; B2, temperature measurement; B3, counseling on danger signs in the newborn; B4, breastfeeding counseling; and B5, observation of breastfeeding. Table 1 shows the question wording from the DHS7 core questionnaire. We did not include newborn care outcomes that were woman/family led (e.g., breastfeeding, prelacteal feeds).

The analysis focused on the postnatal check and 5 health care provider-initiated specific newborn care interventions included in the standard DHS questionnaire.

To be consistent with global indicators for postnatal care, we limited the analysis to the most recent births within 2 years before the survey. We included any postnatal check (pre- or post-discharge) in the first 2 days (Table 1). We excluded newborns who died in the first 2 days of life or who were born in the 2 days before the survey. The sample sizes for each country are shown in Table 2.

All analyses were completed separately by country, adjusting for the complex sampling design, which ensures that each sample is nationally representative, and using the weights provided in the child datasets to account for sampling probability and nonresponse for each survey. We conducted all the statistical analyses using R,^23^ adjusting for the complex sampling design by using the survey package.^24^

## AIM 1: DESCRIBE COVERAGE OF NEWBORN POSTNATAL CARE AND NEWBORN CARE INTERVENTIONS AND GAPS IN QUALITY

### Contact and Content Coverage

First, we present simple coverage of newborn postnatal checks (A) and each of 5 specific newborn care interventions among all newborns (B1 to B5) in the sample defined above for descriptive comparison of differences in coverage. Second, we constructed a co-coverage index of specific newborn care interventions (B) by adding the total number of interventions received among 5 possible provider-initiated interventions, a method similar to Victora et al.^25^ and Carvajal-Aguirre et al.^26^

### Quality-Coverage Gaps

To understand gaps in quality coverage, we analyzed newborns for whom any contact with a health care provider was reported but who did not receive all expected interventions (B1 through B5). We defined the denominator, coverage of any postnatal contact, as whether a woman responded that her baby received a postnatal check (A) or reported at least 1 specific newborn care intervention (B1, B2, B3, B4, or B5). We defined this broadly to reduce the potential that a case was excluded from the denominator due to misinterpretation of the question about a postnatal check by the respondent. We examined quality-coverage gaps using different numerators. First, we describe the proportion of newborns reported as receiving each specific newborn care intervention (B1 through B5) among those with any postnatal contact (A or one of B1 through B5). The quality-coverage gap for each intervention is the difference between any postnatal contact coverage and intervention-specific coverage (100 minus the percentage with intervention coverage). Second, we calculated co-coverage of the 5 specific newborn care interventions (B) among newborns with any postnatal contact (A or one of B1 through B5).

## AIM 2: DESCRIBE INTERNAL CONSISTENCY IN REPORTING POSTNATAL CHECKS AND SPECIFIC NEWBORN CARE INTERVENTIONS

### Internal Consistency in Reporting Contact and Content of Newborn Care

We constructed a variable for internal inconsistency based on the responses to 2 questions—report of a postnatal check (A) and report of the newborn's cord being checked (B1). The postnatal check survey question uses an example of checking the newborn's cord when explaining what a postnatal check is; therefore, a newborn who has had the umbilical cord checked should also be considered to have had a postnatal check. Thus, we calculated the proportion of newborns who had a reported postnatal check (A) among those newborns who were reported as having their umbilical cords checked (B1). This is shown as a proportion of all newborns in the sample with all possible combinations of postnatal check and umbilical check results as shown in Table 3.

**TABLE 3.** Two-Way Table Showing Plausible and Internally Inconsistent Survey Response Possibilities for Postnatal Check and Umbilical Cord Check

		Umbilical Cord Check	
		Yes	No
Postnatal check	Yes	Plausible (coverage)	Plausible (quality-coverage gap)
	No	Internal inconsistency	Plausible (coverage gap)

### Concordance Between Interventions

To determine if any interventions are well represented by measurement of the postnatal check, and better understand the concordance between newborn care interventions and reported postnatal checks, we calculated agreement of responses between pairs of interventions or intervention and postnatal check. This was done by summing the total number of newborns who received both interventions and the number who received neither intervention, divided by the total number of newborns.

### Ethics

The ICF International Institutional Review Board (IRB) conducted an ethical review of all survey tools and protocols, and an IRB in the host country approved each country survey. Interviewers obtained informed consent and ensured voluntary participation before each interview.

Ethical approval to conduct these analyses was granted by King's College London College Research Ethics Committee (LRS-17/18-5570). The project was registered with the King's College London Data Protection Registration (DPRF-17/18-8170), in compliance with European data regulations. We accessed these datasets through a written agreement with the DHS Program.

## RESULTS

Background characteristics of the sample are shown in Supplement Table 1. The proportion residing in an urban area ranged from 9.0% in Burundi to 53.8% in Nepal. Having any education ranged from 26.0% in Guinea to 98.7% in Zimbabwe. Facility birth ranged from 36.0% in Ethiopia to 93.1% in Malawi.

### Coverage of Newborn Care and Quality-Coverage Gaps

**FIGURE 1:**
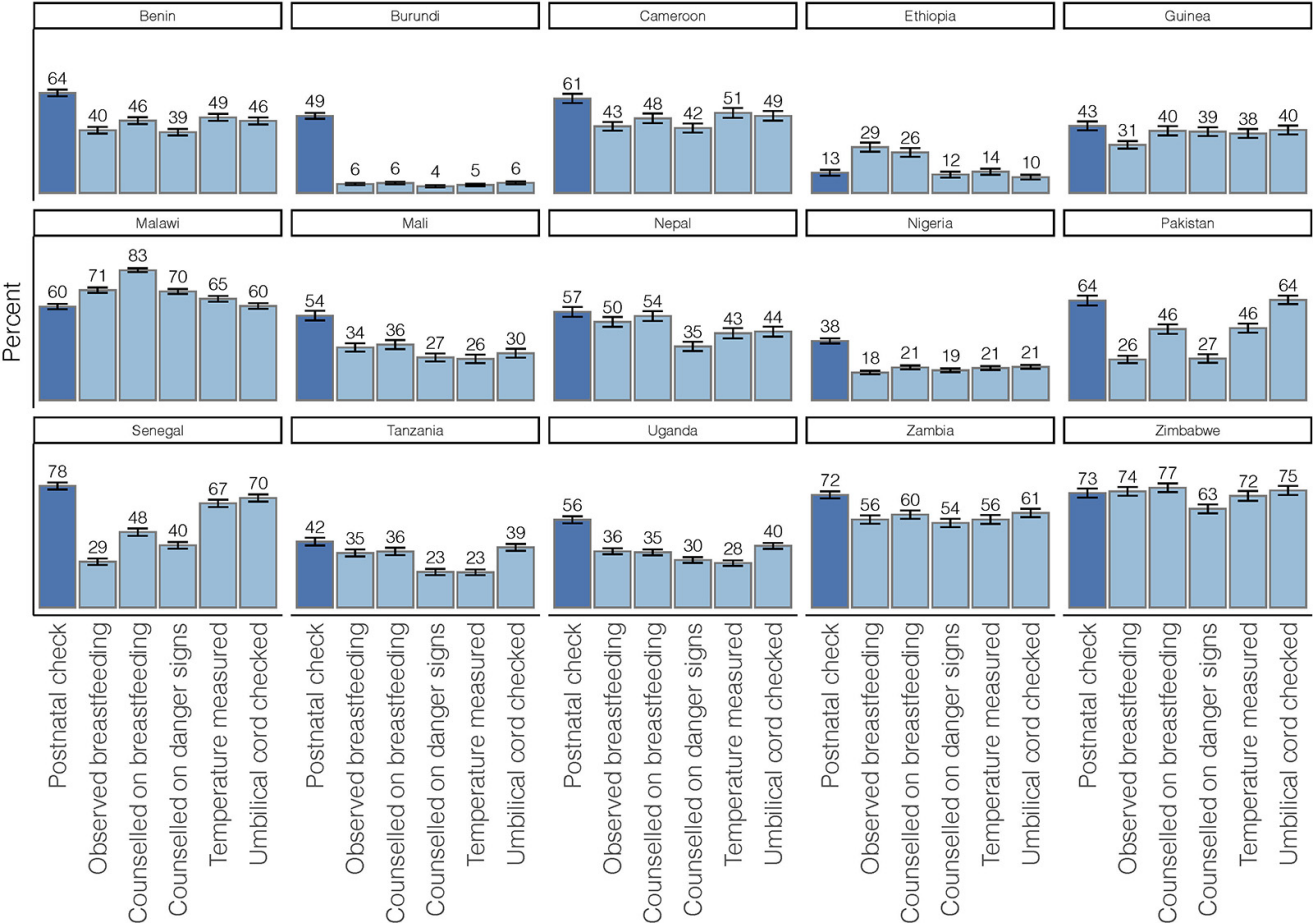
Coverage of Newborn Postnatal Care Expressed as the Proportion of Newborns Receiving Postnatal Checks or Specific Provider-Initiated Newborn Care Interventions Among All Newborns Born in the Sample, by Country

**FIGURE 2:**
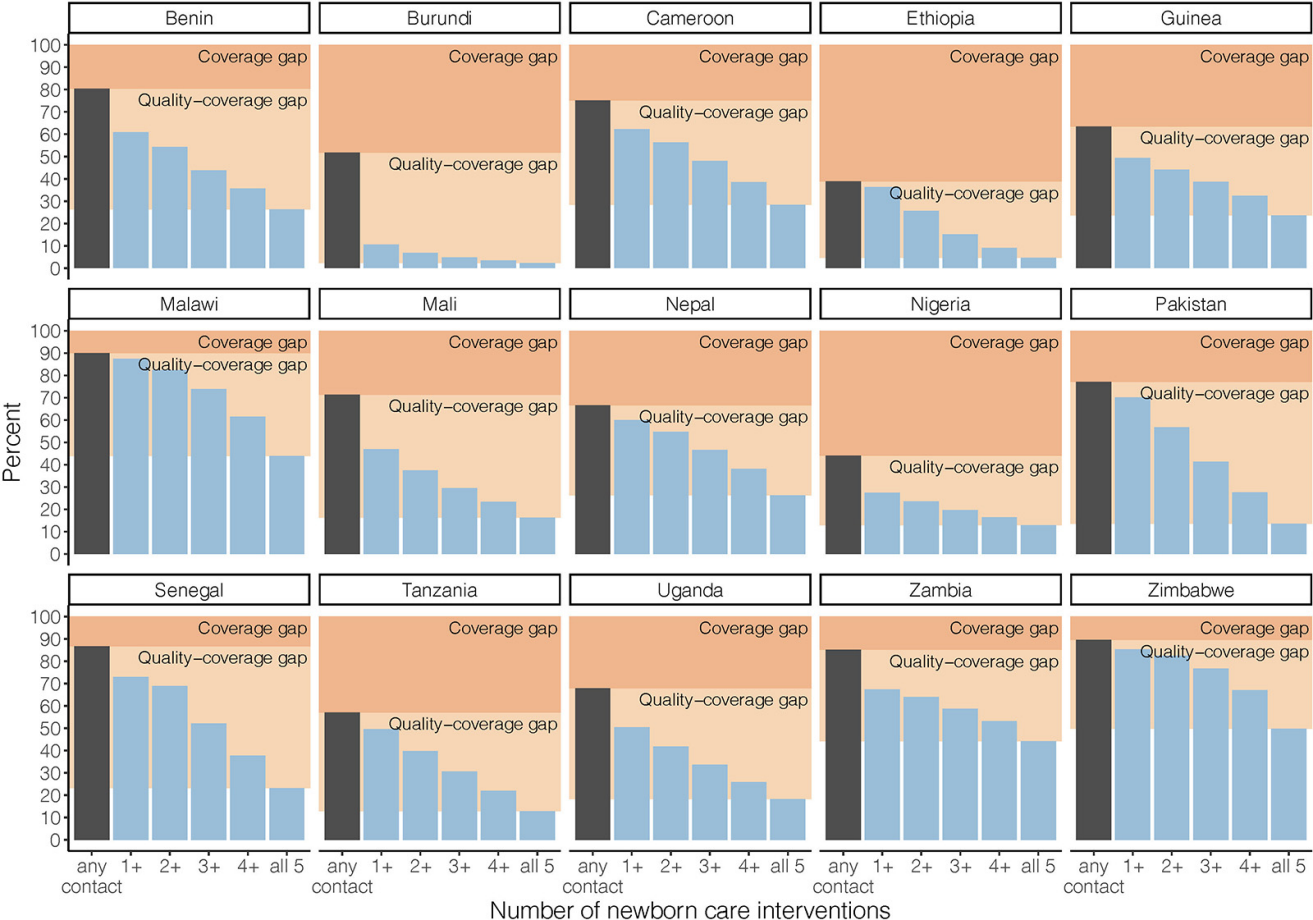
Coverage Cascade for All Newborns Shown as the Proportion With Any Newborn Postnatal Contact (Postnatal Check or Any Specific Intervention) and the Proportion With Each Level of Co-coverage Index Score

Report of a postnatal check within 2 days of birth ranged from 12.9% (95% confidence interval [CI]=11.1,14.9) of newborns in Ethiopia to 78.0% (95% CI=75.7,80.3) in Senegal (Figure 1). Report of specific newborn care interventions varied widely by intervention within countries as well as between countries. For example, in Senegal, while 29.4% of women reported a health care worker observed them breastfeeding, 70.3% reported a health care worker checked the newborn's umbilical cord. Coverage of all specific newborn care interventions was low in Ethiopia and Nigeria, where each of the interventions was reported for less than one-third of newborns. Zimbabwe and Malawi achieved higher coverage of all postnatal interventions of interest with at least 6 of 10 newborns reported as receiving each intervention (Figure 1); however, a co-coverage index score (total number of interventions received) of all 5 interventions was still under 50% (Table 4, Figure 2), even in these 2 countries with the highest coverage (range 0–5 in all countries).

**TABLE 4.** Co-coverage of Provider-Initiated Newborn Care Interventions^a^ in 15 Low- and Middle-Income Countries

Country	Co-coverage (Number of Interventions), %						Mean (SD)
	0	1	2	3	4	5	
Benin	39.2	6.7	10.5	8.1	9.3	26.3	2.2 (2.10)
Burundi	89.3	3.8	2	1.4	1.2	2.2	0.3 (0.96)
Cameroon	37.9	5.8	8.4	9.5	10.2	28.3	2.3 (2.12)
Ethiopia	63.6	10.8	10.5	6.1	4.3	4.7	0.9 (1.46)
Guinea	50.6	5.2	5.5	6.2	8.8	23.7	1.9 (2.15)
Malawi	12.5	5	8.6	12.3	17.6	43.9	3.5 (1.76)
Mali	53	9.6	7.9	6.1	7.1	16.3	1.5 (1.95)
Nepal	40.1	5.1	8.2	8.5	11.9	26.2	2.3 (2.12)
Nigeria	72.6	3.8	4	3.1	3.6	12.9	1 (1.81)
Pakistan	29.9	13.4	15.4	13.8	14	13.6	2.1 (1.80)
Senegal	27.1	4	16.8	14.4	14.5	23.2	2.5 (1.91)
Tanzania	50.3	9.9	9.1	8.6	9.2	12.8	1.5 (1.88)
Uganda	49.6	8.6	8.2	7.7	7.7	18.2	1.7 (2.00)
Zambia	32.6	3.5	5.2	5.6	8.9	44.2	2.9 (2.23)
Zimbabwe	14.7	2.9	5.7	9.6	17.3	49.8	3.6 (1.81)

Abbreviation: SD, standard deviation.

Report of specific newborn care interventions varied widely by intervention within countries as well as between countries.

Coverage gaps for any contact with a health worker in the first 2 days of life ranged from 10.0% in Malawi to 61.2% in Ethiopia (Figure 2). Despite a small coverage gap in Senegal (13.3%), coverage of all 5 interventions was low, at 23.2% (Table 4). The quality-coverage gap was lowest in Nigeria (31.2%), although coverage of any contact with a health care provider was low (44.1%) as was coverage of all 5 interventions (12.9%). The combined coverage and quality-coverage gaps (percentage of newborns without all 5 interventions) ranged from 50.2% in Zimbabwe to 97.8% in Burundi.

**FIGURE 3:**
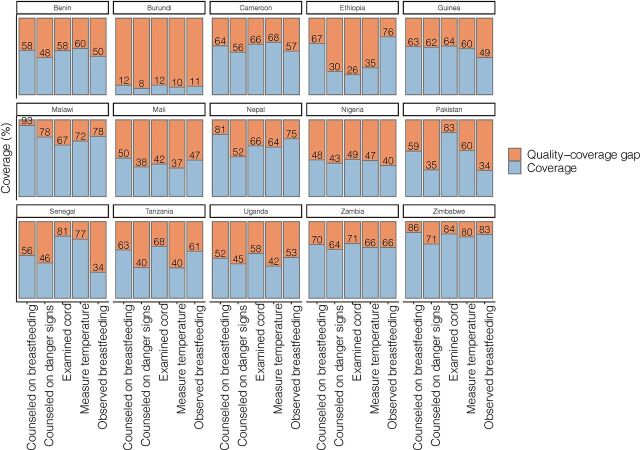
Intervention-Specific Coverage and Quality-Coverage Gaps Among Women Reporting Any Newborn Postnatal Contact (Postnatal Check or Any Specific Intervention) as Percentage Reporting Each Newborn Care Intervention

**FIGURE 4:**
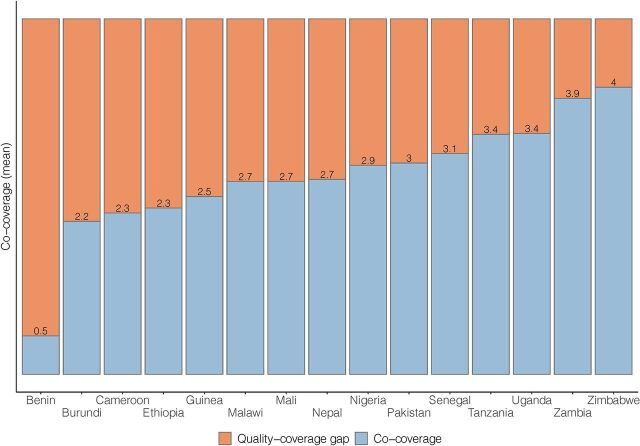
Full Content Quality-Coverage Gap Among Women Reporting Any Newborn Postnatal Contact (Postnatal Check or Any Specific Intervention), Mean Co-coverage of 5 Interventions (Counseling on Breastfeeding, Observing Breastfeeding, Examining Umbilical Cord, Measuring Temperature, and Counseling on Danger Signs)

Among newborns with any postnatal contact (a postnatal check or any specific newborn care intervention), coverage of specific newborn care interventions was not universal (Figure 3). Quality-coverage gaps were highest in Burundi, where individual intervention coverage was not higher than 12%, leaving a quality-coverage gap of >88%. Quality-coverage gaps were lowest (<29%) in Zimbabwe, where individual intervention coverage was >71%. Mean co-coverage ranged from 0.5 interventions in Burundi to 4.0 in Zimbabwe (among newborns with any postnatal contact, Figure 4).

### Internally Inconsistent Responses in Reporting Newborn Postnatal Care

**FIGURE 5:**
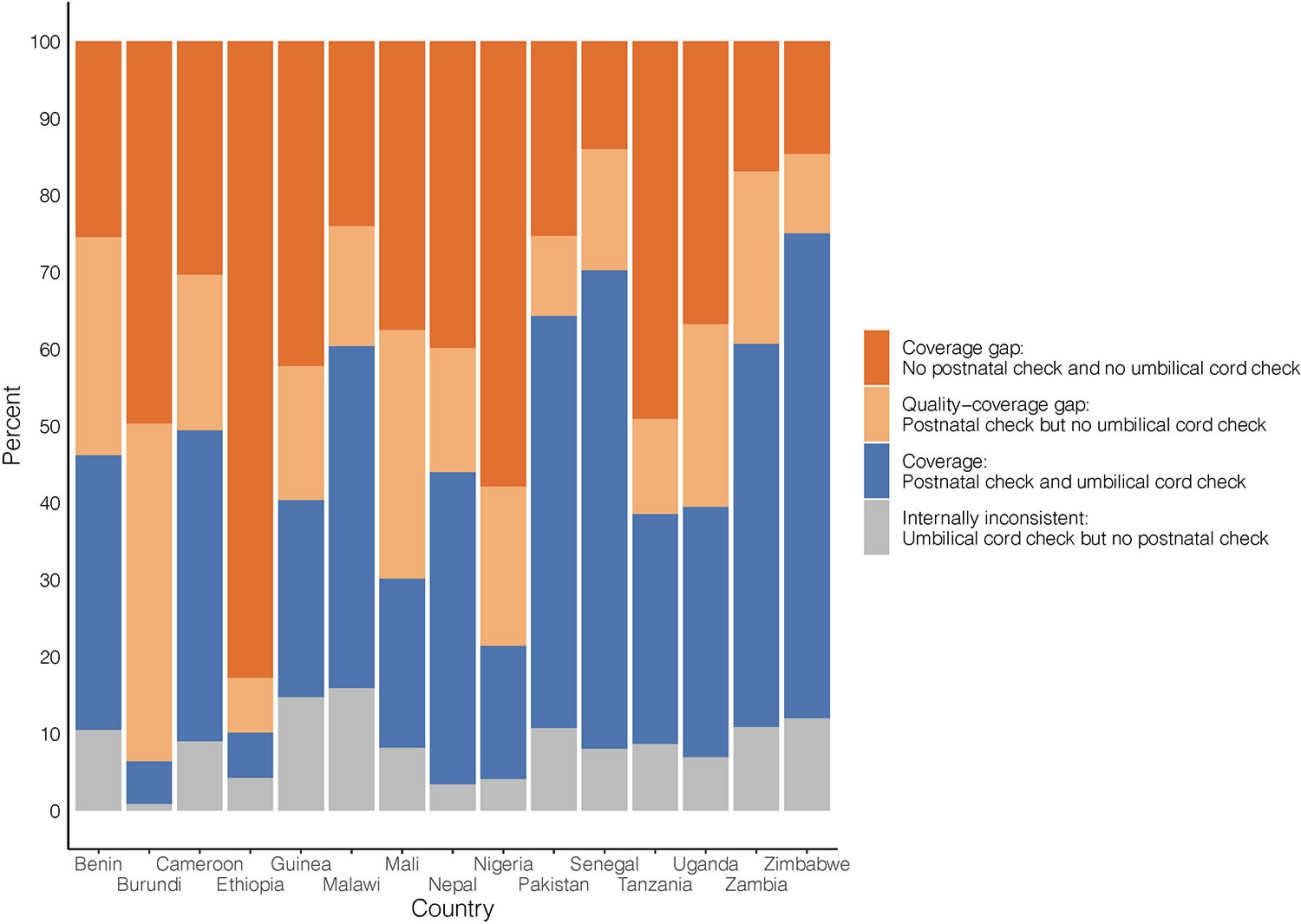
Coverage of Newborn Postnatal Checks and Umbilical Cord Checks, Gaps in Coverage and Quality, and Internal Inconsistencies in Survey Responses

Although the postnatal check survey question includes the example of checking the cord, among those with reported umbilical cord checks, postnatal checks were not universally reported. Internally inconsistent responses (“yes” to a check on the umbilical cord but “no” to a postnatal check) were as high as 15.9% in Malawi (Figure 5). This internal inconsistency was lowest in Burundi (<1%), where coverage of umbilical cord checks was very low (6.5%).

### Agreement Between Postnatal Check and Newborn Care Interventions

**FIGURE 6:**
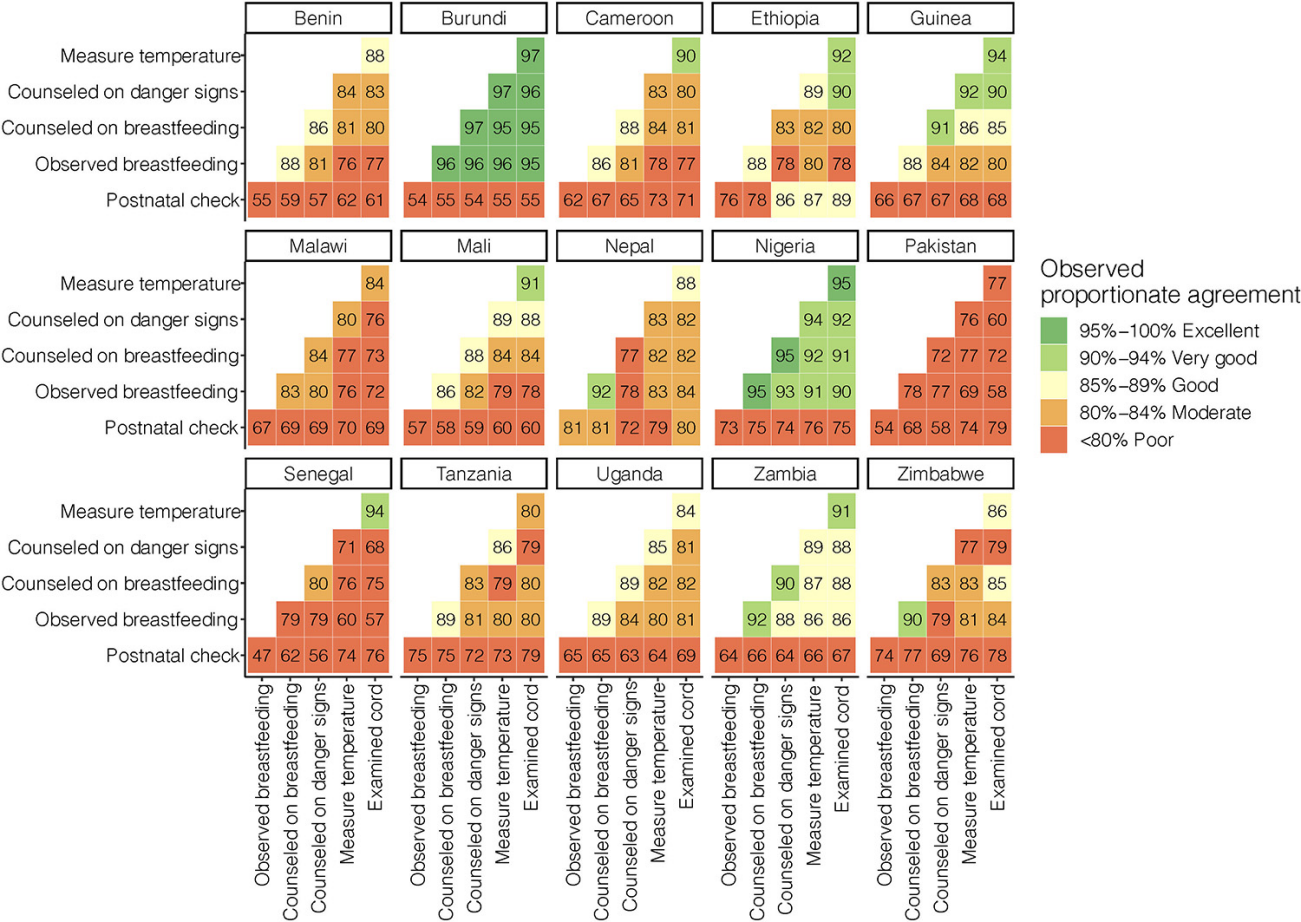
Agreement Between Newborn Care Interventions Expressed as the Number of Cases in Which Newborns Either Received Both Interventions or Received Neither Intervention, Divided by Total Number of Newborns

Agreement between the postnatal check and specific newborn care interventions was low with some variation by intervention and country, ranging from 47% to 89% (Figure 6). Agreement between pairs of newborn care interventions ranged from 57% to 97%. Agreement was high between intervention pairs in Ethiopia and Nigeria (>90%), where coverage of care was consistently low across interventions and survey responses for most interventions was “no.” Agreement was lowest in Pakistan and Senegal, where coverage of some interventions was more than double coverage of other interventions. The intervention of being counseled on breastfeeding had the highest agreement with other newborn care interventions.

## DISCUSSION

These findings highlight a quality-coverage gap for newborn care and discordance between survey indicators for newborn postnatal checks and receipt of specific newborn care interventions. While reported postnatal check was representative of coverage of newborn care interventions in some countries (Guinea, Nepal, Zimbabwe), a postnatal check over- or underestimated coverage of newborn care interventions in other countries (Angola, Burundi, Mali, Nigeria, Senegal).

The findings highlight a quality-coverage gap for newborn care and discordance between survey indicators for newborn postnatal checks and receipt of specific newborn care interventions.

### Challenges in Assessing Newborn Postnatal Care Coverage

Contact coverage for maternal and newborn care declines at each stage along the continuum of care from pregnancy (antenatal care) to birth (skilled attendance) and is lowest for postnatal care.^27^ Where contact coverage occurs, quality-coverage gaps have been noted across this continuum.^16^^,^^27^ Even among women receiving their first antenatal care check in the first trimester and with at least 4 checks during pregnancy, the full suite of recommended interventions is not always reported.^15^^,^^28^ As in our study, a quality-coverage gap for provider-initiated newborn care interventions was found in Nigeria, Ethiopia, and India, where no woman or newborn had received the full recommended content during a postnatal check.^27^ Evidence of a quality-coverage gap is further supported by observational evidence.^29^^–^^32^ However, it is possible a woman reported a postnatal check, remembering a visit at a facility or at home, but could not report specific interventions due to recall or never being informed about the interventions delivered.

Qualitative research in Ghana found that for facility births, newborn checks take place out of the mother's sight, and women were rarely informed about the types of checks being done.^33^ Unclear or poorly defined terms in health surveys can have large or systematic effects on results.^34^ Qualitative research with women regarding their understanding of a “health checkup” has shown that women needed some guidance to understand what it meant to check their baby's health.^19^ Although labor, birth, and antenatal care are well-known and branded concepts, postnatal care does not appear to be understood in the same way.^33^ As such, direct questions about postnatal checks are likely to underestimate coverage. On the other hand, research has shown that women overreport newborn interventions received.^35^ In some settings, the newborn may be kept away from the mother, who is thought to be too tired following birth to participate in newborn care, and care is provided by traditional birth attendants, health workers, or female relatives.^36^^,^^37^ World Health Organization standards for quality of maternal and newborn care in health facilities include the recommendation that women and families receive clear and accurate communication about the care newborns receive.^38^ Such improvements to experience of care around the time of birth may improve accuracy of survey-reported newborn care.

Measurement of postnatal care is complicated. A single provider may be caring for 2 people (the woman and the baby), and checks may occur at multiple time points and in different places (labor ward, postnatal ward, home, outpatient). As Moran et al.^14^ highlighted, beyond a lack of consensus on when the postnatal period begins, operationalizing a cutoff for postnatal care measurement in household survey questions would remain a challenge. When postnatal checks reported in the first hour are considered as solely intrapartum care, postnatal care coverage levels drop considerably.^39^ Additionally, qualitative interviews with women in Tanzania have shown that women did not differentiate between postnatal care and the Expanded Programme on Immunisation (covering vaccination up to or beyond 1 year of age).^40^ To improve the validity of the postnatal check question, as referred to earlier, an example of a postnatal intervention (checking of the umbilical cord) was added to the DHS questionnaire following recommendations from qualitative research on women's understanding of the postnatal check question.^19^^,^^21^^,^^41^ Although the report of a postnatal check was higher for newborns who had a reported umbilical cord check compared with those who did not have a cord check, we still found inconsistency between reported postnatal checks and reported umbilical cord checks. A woman who reports that her newborn received an umbilical cord check could also be expected to report a postnatal check, as an umbilical cord check was used as an example of a postnatal check. Yet this study shows that this was not always the case. Further research is needed to explore the reason behind this discrepancy, including comprehension of the question or confusion regarding the timeline. The latest version of the DHS questionnaire has updated the wording for the postnatal check survey question, “Now I would like to talk to you about checks on (NAME'S) health—for example, someone examining (NAME), checking the cord, or talking to you about how to care for (NAME).”^42^

DHS translates questionnaires into primary languages for each country using back-translation and pretests them to ensure they are understandable to women.^43^ Questionnaires are not officially translated further into less widely spoken languages or languages without a written script. Instead, interviewers are instructed to modify the wording of questions to fit local dialects and culture without changing the meaning of the question^44^; however, some aspects of the postnatal care questions may be lost in translation. In Malawi, where internally inconsistent reporting of newborn care was highest, most interviews were conducted in Chichewa using a Chichewa questionnaire, and <1% of interviews included in the analysis were conducted in a language different from the questionnaire itself. Conversely, in Guinea, the questionnaires were all in French, while the interviews were conducted largely in Soussou, Peul, and Malinke among others.

### Challenges in Assessing Quality of Newborn Postnatal Care

As evidenced by this study, a “health check” is not necessarily reflective of the level of specific newborn care interventions received in a given country due to substantial quality-coverage gaps in many countries and wide differences in coverage of various newborn care interventions. While coverage of newborn care interventions was higher among newborns receiving a postnatal check, examining only this as an indicator ignores the substantial number of newborns who received provider-initiated newborn care interventions but whose mothers did not report a postnatal check. Some women may believe postnatal checks are only required for sick newborns.^45^ Research has shown women know checks had occurred because they were asked a question (e.g., on breastfeeding) or when equipment (e.g., thermometer) was used.^33^

A “health check” may not reflect the actual level of specific newborn care interventions received.

Previously, the Every Newborn Action Plan Measurement Improvement Roadmap proposed using early breastfeeding as a tracer indicator for essential newborn care^46^; however, research has shown that this indicator did not correlate highly with other elements of essential newborn care besides skin-to-skin contact in 1 study.^47^^,^^48^ While this study focused on postnatal care and only included provider-initiated interventions, agreement between postnatal check and other interventions was low for most interventions in most countries. This result may be due to inconsistent survey responses (not reporting a postnatal check but reporting specific newborn care interventions) or a result of poor quality of care and the quality-coverage gap (a check occurred but complete care was not provided). We found counseling on breastfeeding had slightly higher agreement with a postnatal check than other newborn care interventions although other research has shown counseling interventions to have lower validity in surveys than those reflecting physical examination.^35^ Additionally, counseling interventions during antenatal, family planning, and sick child care are commonly overreported.^49^ As such, measuring survey reports of observation of breastfeeding may be more important than counseling on breastfeeding.

While tracking content coverage and quality could be done with specific individual tracer indicators (measuring 1 intervention to estimate the coverage of multiple interventions or quality coverage), this article, in addition to Sitrin et al.,^47^ shows that this is not likely to be useful given such low agreement. Quality newborn care could instead be estimated with effective coverage measures. Effective coverage, which draws from quality metrics, is defined as the proportion of a population in need of a service that receives services from a facility equipped to provide care (input-adjusted effective coverage) or receives services in line with quality standards (quality-adjusted), or when health outcomes are gained (outcome-adjusted).^11^^,^^12^ For postnatal care, due to difficulties in attributing neonatal mortality to specific services, Marsh et al.^11^ recommended measuring quality-adjusted effective coverage—which encompasses timely and appropriate response and respectful care and treatment. While effective coverage is an important and valuable concept, it can be challenging to measure because it usually involves combining data from different sources, possibly measured at different times,^11^ and facility data related to postnatal care are limited in some settings or may not be commonly publicly available.^50^ Further, while input-, quality-, or outcome-adjusted measures of effective coverage are useful for national monitoring, they are not typically measured at the individual level.

Quality newborn care could be estimated with effective coverage measures.

Conversely, co-coverage is measured at the individual level, only requires data from a single source, and may be more accessible to data users than effective coverage measures, which can involve linking multiple datasets as described above.^11^ Co-coverage can more specifically show the proportion of the population receiving most or all of the recommended postnatal care interventions, providing a more granular understanding of gaps in care provision to better inform service development needs.^51^ Furthermore, co-coverage can be useful for equity analyses to identify high-risk groups lagging behind^25^^,^^51^^–^^54^ and may be particularly advantageous when used to replace multiple coverage estimates in multicountry or time trend analyses.^16^^,^^51^^,^^55^ However, country-specific questionnaire adaptations or differences may inhibit cross-country comparison. It is worth noting that this measure of co-coverage can only be used to summarize interventions for healthy or full-term newborns, which may preclude special interventions for small and sick newborns.

### Strengths and Limitations

This study is one of the first to look beyond the quality-coverage gap and examine internal consistency between newborn care interventions and postnatal checks. It adds to our understanding of postnatal care measurement and the reliability of the questions that provide essential global monitoring information for health service improvement.

Study results add to the understanding of postnatal care measurement and the reliability of the questions that provide essential global monitoring information for health service improvement.

Although this study included data from 15 nationally representative surveys covering a range of geographic regions and care coverage levels, limitations should be noted. These analyses were based entirely on self-report from women about the care their newborns received after birth. Validation studies for some of these indicators have been conducted previously, producing inconsistent findings.^18^^,^^56^^–^^58^ For example, in Kenya and Swaziland, interventions such as counseling on breastfeeding and counseling on danger signs in the newborn met criteria for individual-level accuracy and low population-level bias, whereas other interventions such as examining the baby did not meet these criteria.^57^ A recent large validation study on survey measurement of maternal and newborn indicators did not validate the interventions in this analysis but did show limited potential for content of care indicators in surveys.^59^ While the internally inconsistent responses discussed in this article may relate to women's understanding of the survey questions or the care their newborns received, inconsistencies may also be related to inter-interviewer differences. Future research may use DHS fieldworker questionnaires to examine data consistency in relation to worker characteristics. Additionally, recent changes in the postnatal check question wording may improve question understanding in future surveys; however, it may limit comparability of questions for trend analysis.^42^

Furthermore, when women reported receiving a postnatal check for their newborn but not any of the newborn care interventions in this study, their report may be accurate if they received interventions beyond the 5 we considered. To limit the effect of this bias, where possible we limited the analysis on internal consistency to the specific situation cases in which women reported their newborns as receiving a cord check but reported no newborn postnatal check, as checking the cord is an example in the newborn postnatal check question.

## CONCLUSION

Prompt, safe, and high-quality postnatal care is vital for improving newborn survival. Reliable and standardized measurement of content of care is essential to drive improvements in coverage and quality. In low- and middle-income country surveys, we found coverage of newborn care varied widely by intervention, making a single question about receipt of a health check or a tracer indicator a poor proxy for coverage of comprehensive newborn care. To improve global measurement and tracking of postnatal care, collecting information on content of care is critical. While facility data and effective coverage measures may identify bottlenecks in service provision that can be used to improve quality of care, co-coverage measures are useful for program managers to understand content coverage and show what proportion of the population is receiving all or most important interventions for newborn postnatal care. Use of co-coverage measures will allow for additional analysis of survey data, particularly of equity in coverage and care to identify high-risk groups lagging behind. Additionally, as each year more births occur in health facilities, investment in and use of routine data systems can complement surveys to track content of newborn care.

## Supplementary Material

GHSP-D-21-00209-supplement.pdf

## Notes

**Peer Reviewed**

**First published online:** November 5, 2021.
