# Supplementary material for: Household Survey Measurement of Newborn Postnatal Care: Coverage, Quality Gaps, and Internal Inconsistencies in Responses
Source: Glob Health Sci Pract. 2021 Dec 31;9(4):737–51. doi: 10.9745/GHSP-D-21-00209 (PMC8691891; doi:10.9745/GHSP-D-21-00209)
Supplement: GHSP-D-21-00209-supplement.pdf [file GHSP-D-21-00209-supplement.pdf]

Supplement to: Peven K, Day LT, Bick D, et al. *Household survey measurement of newborn postnatal care: coverage, quality gaps, and internal inconsistencies in responses. Glob Health Sci Pract*. 2021;9(4). https://doi.org/10.9745/GHSP-D-21-00209

Supplement. Table 1 Background characteristics of the sample

	**Urban residence**	**Any education**	**Mother's age at birth (years)**			**First live birth**	**Wealth quintile**					**Facility birth**
			<20	*20-34*	*35+*		*Poorest*	*Poorer*	*Middle*	*Richer*	*Richest*	
Benin	38.4	36.9	13.2	73.1	13.7	22	21.1	20.6	20.7	19.8	17.9	85
Burundi	9	56.3	7.5	73.8	18.7	17.3	22.1	22.1	20.6	18.8	16.3	85.4
Cameroon	43.7	73.3	19.4	68.5	12.1	23.8	22.3	23.4	20.9	18.4	14.9	67.8
Ethiopia	12.1	38.8	11.9	72.6	15.5	20.6	23.5	22.1	20.7	18.2	15.5	36
Guinea	28.7	26	19.4	65.1	15.5	19.2	23.6	22.7	19.5	18.6	15.6	54.5
Malawi	13.7	87.1	20.7	67.2	12.1	27.5	25.3	22.7	19.3	16.9	15.8	93.1
Mali	21.1	29.2	16.6	67.4	16	17.5	20.1	21.6	21.2	19.1	17.9	69.8
Nepal	53.8	70.3	22.7	73.5	3.8	40.7	20.9	20.9	23.1	20.6	14.5	64.3
Nigeria	38.5	55.2	12.7	71.4	15.9	18.9	21.6	22.9	20.5	18.6	16.5	40.4
Pakistan	32.9	52.8	9	80.1	10.8	23.7	21.4	18.8	21.7	18.8	19.3	71.3
Senegal	35.9	39.8	13	69.9	17.1	24.6	24.4	22.4	20.7	17	15.5	80.2
Tanzania	27.7	80.4	18.7	65.8	15.5	27.1	24.4	20.9	18.7	19	16.9	64.9
Uganda	21.4	89.9	17.3	69.8	12.8	22.6	22.4	21.2	19	17.6	19.9	76.5
Zambia	34.2	89.7	21.3	63.4	15.4	25.8	25.8	22.5	18.8	17.1	15.9	86.3
Zimbabwe	28	98.7	18.2	70.4	11.4	26.4	24.9	20.4	18	22.3	14.3	81.1
